# A synthetic furanone potentiates the effect of disinfectants on *Salmonella* in biofilm

**DOI:** 10.1111/j.1365-2672.2009.04495.x

**Published:** 2010-03

**Authors:** LK Vestby, J Lönn-Stensrud, T Møretrø, S Langsrud, A Aamdal-Scheie, T Benneche, LL Nesse

**Affiliations:** 1 Section of Bacteriology, National Veterinary InstituteOslo, Norway; 2 Faculty of Dentistry, Department of Oral Biology, University of OsloOslo, Norway; 3 Nofima matAas, Norway; 4 Faculty of Chemistry, Department of Chemistry, University of OsloOslo, Norway

**Keywords:** benzalkonium chloride, biofilm, disinfectant, feed, furanone, hypochlorite, persistence, *Salmonella*

## Abstract

**Aims:**

To study a possible effect of a synthetic brominated furanone on biofilm formation and biofilm resistance to disinfectants in *Salmonella enterica*.

**Methods and Results:**

The effect of a synthetic furanone on biofilm formation of *Salm. enterica* serovar Agona and *Salm. enterica* serovar Typhimurium (11 strains of different origins) was evaluated in a microtiterplate assay. A significant reduction in biofilm build-up in microtiterplates by the furanone was observed for seven of the strains tested. Biofilms by two *Salm.* Agona feed factory strains and the effects on survival after exposures to disinfectants (hypochlorite and benzalkonium chloride) were assessed for both strains. Pretreatment with furanone significantly potentiated the effect of the two disinfectants for both strains.

**Conclusions:**

The effect of disinfectants on *Salmonella* in biofilm was significantly enhanced when the biofilm was grown in the presence of furanone. This was probably because of an effect on biofilm architecture, composition and in some cases also biofilm build-up.

**Significance and Impact of the Study:**

The present study gives valuable new knowledge in the fight against *Salmonella* biofilm in the environment because of the potentiated effect of conventional disinfectants.

## Introduction

*Salmonella* is an infectious agent involved in millions of cases of human disease all over the world (http://www.who.int/mediacentre/factsheets/fs139/en/index.html). Contaminated feed and feed ingredients with *Salmonella* is a well-known problem and several authorities, as well as the feed industry, are using large resources in the fight against *Salmonella* (Veldman *et al.* 1995; Davies and Wray 1997; Lunestad *et al.* 2007; Anon. 2008). One way for *Salmonella* to persist in the feed factory environment is in a biofilm (Vestby *et al.* 2009).

‘Biofilms are defined as matrix-enclosed bacterial populations adherent to each other and/or to surfaces or interfaces’ (Costerton *et al.* 1995). In a biofilm, *Salmonella* is known to be less sensitive to disinfectants than their planktonic counterparts (Scher *et al.* 2005; Møretrø*et al.* 2009).

Quorum sensing has been shown to play a role in biofilm formation and maturation in several bacterial species (Davies *et al.* 1998; Costerton *et al.* 2007). The Autoinducer-2 (AI-2) quorum sensing system is a widespread quorum sensing system and was first described in *Vibrio harveyi* as being involved in bioluminescence (Walters and Sperandio 2006) and later in *Salmonella* (Surette and Bassler 1998). In this system, the LuxS enzyme converts *S*-ribosyl-homocysteine into homocysteine and 4,5-dihydroxy-2,3-pentanedione (DPD). DPD spontaneously cyclizes to form different AI-2 signalling molecules (Schauder *et al.* 2001). Although earlier studies indicate that the *luxS* gene may play a role in biofilm production by *Salmonella*, the importance of AI-2 in this genus is uncertain (Prouty *et al.* 2002; De Keersmaecker *et al.* 2005).

Halogenated furanones were first discovered to inhibit bacterial colonization of the red algae *Delisea pulcra* (Kjelleberg and Steinberg 2001) and have also been shown to act as biofilm inhibitors for several bacterial species in laboratory experiments (Hentzer *et al.* 2002; Rice *et al.* 2005; de Nys *et al.* 2006; Lönn-Stensrud *et al.* 2007). Furanones have been suggested to interfere with the quorum sensing system in Gram-negative bacteria (de Nys *et al.* 2006). The synthetic brominated furanone used in the present study has previously been shown to have an inhibitory effect on biofilm formation in oral streptococci and is reported to interfere with the AI-2 quorum sensing system in oral streptococci and *Staphylococcus epidermidis* (Lönn-Stensrud *et al.* 2007; Benneche *et al.* 2008; Lönn-Stensrud *et al.* 2009). Previous studies have shown that susceptibility to some antibiotics was increased when used in combination with furanones (Hentzer *et al.* 2003; Bjarnsholt *et al.* 2005; Janssens *et al.* 2008).

The aim of this study was to evaluate the effect of a synthetic brominated furanone on *Salmonella* in biofilm. Investigation on preexposure of this furanone followed by treatment with conventional disinfectants used in the feed industry was conducted to evaluate the potential use of this combination as a weapon in the fight against *Salmonella*. As the furanone used in this study is reported to interfere with the AI-2 quorum sensing system, the effect of this quorum sensing system on biofilm formation in *Salmonella* was studied.

## Materials and methods

### Bacterial strains and culture conditions

A total of 11 *Salmonella enterica* strains of serovar Agona and serovar Typhimurium were used in this study (Table 1), including strains from feed and fish meal factories and clinical isolates as well as two reference collection strains. All isolations were carried out at different private or official laboratories and verified at the National Reference Laboratory for *Salmonella* in Feed and Food at the National Veterinary Institute.

**Table 1:** Bacterial strains and strains used in this study

Strain	Serovar	Origin	Genotype
2168-2	*Salmonella enterica* serovar Agona	Human (clinical)	Wild type
2168-6	*Salm.* Agona	Human (clinical)	Wild type
2168-10*	*Salm.* Agona	Feed factory	Wild type
71-4*	*Salm.* Agona	Feed factory	Wild type
71-5*	*Salm.* Agona	Feed factory	Wild type
71-3*	*Salm.* Agona	Feed factory	Wild type
5315*	*Salmonella enterica* serovar Typhimurium	Feed factory	Wild type
112-6*	*Salm.* Typhimurium	Feed factory	Wild type
ATCC 14028	*Salm.* Typhimurium	Reference collection	Wild type
ATCC 700720D (LT2)	*Salm.* Typhimurium	Reference collection	Wild type
LT-2-3819	*Salm.* Typhimurium	*Salmonella* genetic stock centre, University of Calgary	ygaG101::Mud J (*luxS*)

* Vestby *et al.* (2009).

All strains were stored at −80°C in Brain Heart Infusion broth (Difco, BD, Franklin Lakes, NJ, USA) supplemented with 15% glycerine (Merck KGaA, Darmstadt, Germany) and recovered on blood agar (sheep blood) at 37·0 ± 1·0°C overnight. The bacterial cultures were then transferred into Luria Bertani broth (LB; Merck) and incubated statically overnight at 37·0 ± 1·0°C to obtain an overnight working culture.

### Crystal violet colorimetric assay (biofilm)

Biofilm formation was measured as previously described with slight modifications (Woodward *et al.* 2000; Vestby *et al.* 2009). In short, an overnight culture of *Salmonella* was diluted in LB broth without NaCl (LB ^wo^/NaCl; bacto-tryptone 10 g l^−1^, yeast extract 5 g l^−1^) to OD_595_ = 0·2, and 15 μl was transferred to each well in a 96-well polystyrene microtiterplate (Nunc, Nuncleon, Roskilde, Denmark) containing 100 μl LB ^wo^/NaCl. The plates were incubated at 20·0 ± 1·0°C for 2 days. OD_595_ was measured to assess planktonic growth before the plates were emptied and gently washed once with sterile distilled water. The biofilm was stained by adding 130 μl of 1% crystal violet (Sigma-Aldrich, St Louis, MO, USA) and incubated for 30 min in room temperature. The plates were gently washed three times with sterile distilled water to remove excess dye. One hundred and thirty microlitres of ethanol/acetone (70 : 30 w/w) was added and incubated for an additional 10 min in room temperature before OD_595_ was measured.

### Disinfectants

Disinfectants used in this study were 0·05% hypochlorite (Klorin; Lilleborg AS, Oslo, Norway) and 0·02% benzalkonium chloride (Norwegian Pharmaceutical Depot, Oslo, Norway). Both disinfectants were diluted in sterile distilled water to obtain the user concentrations recommended by the manufacturers.

### Furanone

**Figure 1:**
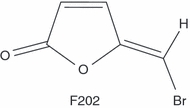
Chemical structure of (*Z*)-5bromomethylene-2(5*H*)-furanone.

The brominated furanone tested was F202 (Fig. 1). The furanone was synthesized at the University of Oslo, Department of Chemistry, as previously described (Benneche *et al.* 2006). The furanone was dissolved and diluted in absolute ethanol to a final stock concentration of 60 mmol l^−1^ and stored at −20·0 ± 1·0°C.

### Effect of furanone on biofilm formation

The crystal violet colorimetric assay was used to asses the effect of furanone F202 on biofilm formation of *Salmonella*. The furanone was first diluted in sterile distilled water to working solutions of 60 and 600 μmol l^−1^ and further diluted to final concentrations of 3, 12 or 48 μmol l^−1^ in the wells using LB ^wo^/NaCl. The same volume of sterile distilled water was added to the negative controls. Triplicates of each sample were used and the experiment was repeated at least two times on different days and with freshly prepared solutions.

To test if addition of furanone affected planktonic growth, OD_595_ was measured before the addition of crystal violet and OD_595_ values of bacterial growth in wells with and without furanone were compared. At least two independent experiments were performed using triplicate samples.

### Antimicrobial effect of furanone and disinfectants

The effect of preexposure to furanone F202 on survival of biofilm grown cells exposed to disinfectants was tested. Two strains with different responses to furanone in microtiterplates (2168-10 and 71-3) were chosen. Overnight cultures were inoculated (1 μl) in sterile centrifuge tubes (Greiner bio-one GmbH, Frickenhausen, Germany) containing 10 ml LB ^wo^/NaCl with 48 μmol l^−1^ furanone or equal volume of distilled water (control). An autoclaved microscope slide (76 × 26 mm; Menzel GmbH + CoKG, Braunschweig, Germany) was placed in each tube. The tubes were incubated at 20·0 ± 1·0°C for 2 days. During incubation, biofilm was formed on both sides of the microscope slide in the liquid–air interface. The slides were washed in sterile saline before drying in room temperature in a safety hood for *c.*1 h. The bactericidal effect of disinfectants was tested by immersing slides (both pretreated or not with furanone) in 15 ml disinfectant solution for 5 min at 20·0 ± 1·0°C followed by immersion in a 20-ml Dey/Engley neutralising broth (Difco). Controls were treated in the same way, but with sterile saline. The biofilm was thoroughly scraped using a sterile cell scraper (BD Falcon, Bedford, MA, USA) and transferred to reagent tubes containing 4 ml sterile saline and about 30 (3 mm) sterile glass beads. The biofilm was disrupted by vortexing at maximum speed for 40 s before parallel serial dilution (Scher *et al.* 2005) and plating onto blood agar (sheep blood). The plates were incubated overnight at 37·0 ± 1·0°C to enumerate the number of CFU. The results were verified in three independent experiments on different days and with freshly prepared solutions using duplicate samples. Results were calculated as the difference between living bacteria exposed to disinfectants (and sterile saline for control) alone and combined with furanone using log_10_-transformed values.

### Bioluminescence assay (AI-2 measurements)

The bioluminescence assay was conducted as previously described (Surette and Bassler 1998; Schauder *et al.* 2001; Lönn-Stensrud *et al.* 2007) with slight modifications. Briefly, cell-free supernatants from the 11 strains of *Salmonella* were prepared by incubating *Salmonella* to late exponential phase (determined by growth curves under the same conditions) in LB ^wo^/NaCl at 20·0 ± 1·0°C followed by centrifugation (Biofuge 17RS, Heraeus- Sepatech, Osterode, Germany) at 867 ***g*** for 10 min. The supernatants were filtered (0·2 μm filters; Whatman GmbH, Dassel, Germany) and stored at −20·0 ± 1·0°C. The second overnight culture of reporter strain *V. harveyi* BB170 was diluted 1 : 5000 in fresh bioluminescence assay medium (Bassler *et al.* 1993). Filtered supernatants of *Salmonella* to be tested were added to a final concentration of 10%. Bioluminescence induction was followed during 6 h in a Synergy HT Multi-Detection Microplate Reader (Biotek, Winooski, VT, USA). Six parallels were used in the bioluminescence assay, and the assay was performed twice at different days and with freshly prepared solutions. The AI-2 levels are given as a fraction of AI-2 activity from reporter strain BB170.

### Statistical analysis

For assessing the effect on *Salmonella* in biofilm by preexposure to furanone F202 followed by treatment with conventional disinfectants, *P-*values for all relevant combinations were tested using paired *t*-test on log_10_-transformed values. For other calculations, statistical methods used are given under results.

Statistical analysis were performed using jmp (ver. 5.0.1a; SAS Institute Inc., Cary, NC, USA).

## Results

### Effect of furanone on biofilm formation

**Figure 2:**
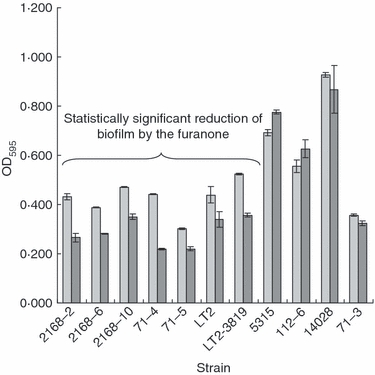
Effect of furanone on biofilm formation by *Salmonella* grown without and in the presence of 48 μmol l^−1^ furanone. Results are given as mean OD_595_ levels for each strain with standard error of the mean. (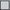) without furanone and (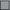) with furanone.)

**Figure 3:**
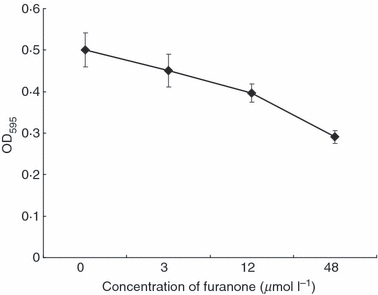
Effect of different concentrations of furanone on biofilm formation in microtiterplates. Only strains with significant reduction in biofilm formation by 48 μmol l^−1^ furanone are included. Results are given as mean OD_595_ levels of all strains with standard error of the mean.

Seven of the strains displayed a statistically significant reduction in biofilm mass in microtiterplates when grown in the presence of 48 μmol l^−1^ furanone as compared to the control (Fig. 2, *t*-test, *P* < 0·05). A reduction in biofilm mass in microtiterplates was also observed for the same strains using concentrations of 3 and 12 μmol l^−1^ furanone (Fig. 3). With a concentration of 12 μmol l^−1^, the reduction was statistically significant (*t*-test, *P* < 0·05), while with 3 μmol l^−1^, the furanone effect was less evident (*t*-test, *P* = 0·35). Addition of furanone had no effect on planktonic growth at the concentrations tested.

### Effect of furanone and disinfectants on *Salmonella* in biofilm

**Figure 4:**
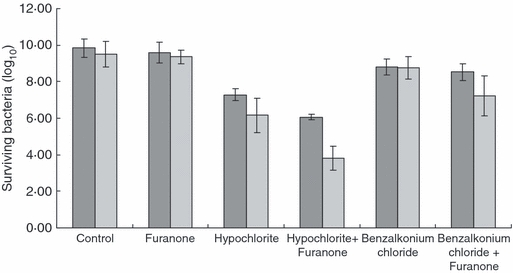
Effect of furanone and disinfectants. Results are given as the mean log_10_-transformed number of colony-forming units recovered on blood agar with standard error of the mean. (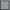, 71-3 and 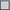, 2168-10 ).

Two *Salm*. Agona strains (2168-10 and 71-3) were used to assess the effect of preexposure to furanone F202 before the use of two conventional disinfectants (hypochlorite and benzalkonium chloride) at recommended user concentrations (Fig. 4). The two strains were selected because of a statistically significantly different effect of the furanone on biofilm build-up in microtiterplates (*t*-test, *P* < 0·05). Based on three independent experiments using two parallels of each variable, a statistically significant effect of preexposure to furanone before disinfection with hypochlorite (paired *t*-test, *P* < 0·05) and benzalkonium chloride (paired *t*-test, *P* < 0·05) was found for both strains. Preexposure to furanone F202 for strain 2168-10 resulted in a 5·7 log_10_ mean reduction in CFU after exposure to hypochlorite compared to a mean reduction of 3·4 log_10_ for exposure to hypochlorite only. A less evident, yet statistically significant, effect of the furanone was observed for 71-3. Results showed a 3·8 log_10_ mean reduction in CFU after exposure to hypochlorite with preexposure to furanone compared to a mean reduction of 2·4 log_10_ for exposure to hypochlorite only. A similar result was obtained by subsequent exposure to benzalkonium chloride after furanone treatment. Results showed a 0·8 and 1·0 log_10_ mean reduction in CFU recovered from biofilm exposed to benzalkonium chloride only, while exposure to benzalkonium chloride after biofilm formation in the presence of furanone F202 resulted in a 2·3 and 1·3 log_10_ mean reduction for strains 2168-10 and 71-3 respectively (Fig. 4). The potentiated effect of both hypochlorite and benzalkonium chloride were statistically significant higher for 2168-10 than for 71-3 (*t*-test, *P* < 0·05 for both disinfectants). No statistically significant effect of furanone on viable bacterial counts (CFU) in the biofilm was observed for either 2168-10 (paired *t*-test, *P* = 0·38) or 71-3 (paired *t*-test, *P* = 0·31).

### The AI-2 system and biofilm formation

**Figure 5:**
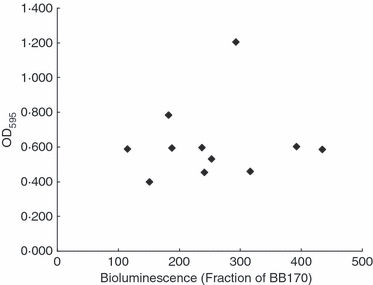
Correlation between biofilm formation in microtiterplates and AI-2 levels. The biofilm results are given as OD_595_ values, and AI-2 levels are given as fraction of AI-2 activity from reporter strain BB170.

**Figure 6:**
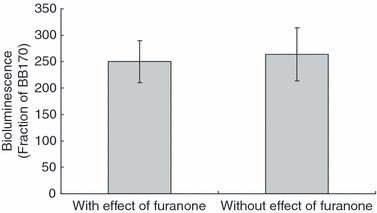
Mean levels of AI-2 activity by strains with and without effect of furanone on biofilm build-up in microtiterplates.

The level of AI-2 in cell-free supernatants did not correlate to the level of biofilm formation in microtiterplates nor indicate an AI-2 threshold level for stimulation of biofilm build-up (Fig. 5). Furthermore, there was no significant difference in biofilm formation by ATCC LT2 and *luxS* inactivated mutant LT2-3819 (*t*-test, *P* = 0·11). The mean level of biofilm mass in microtiterplates (OD_595_) was for ATCC LT2 found to be 0·40 (SE = 0·04) and for mutant LT2-3819 it was found to be 0·54 (SE = 0·04). Whether or not the furanone had an effect on biofilm build-up in microtiterplates was not influenced by the level of AI-2 (*t*-test, *P* = 0·83) (Fig. 6).

## Discussion

In the present study, we have shown that pretreatment with a synthetic brominated furanone before disinfection with conventional disinfectants, like hypochlorite and benzalkonium chloride, resulted in a significant increase in the lethal effect of the disinfectants. To the best of our knowledge, this is the first study that describes the effect of combining brominated furanones with disinfectants on *Salmonella* mediated biofilms. This is important new knowledge because of the fact that most conventional disinfectants alone have limited efficacy against *Salmonella* biofilms from the feed industry (Møretrø*et al.* 2009). According to the European surface test (EN 13697), a reduction of 4 log_10_ or more is required for surface attached bacteria in order to rate a disinfectant as effective (Anon. 2001). In our test, neither of the two disinfectants met this requirement despite the relatively large volumes of disinfectants used. However, combined with furanone, the log_10_ reductions for 2168-10 and 71-3 were 5·7 and 3·8 respectively for hypochlorite. The combination of furanone and hypochlorite met the requirement of the European surface test for 2168-10 and was very close to meeting this requirement also for 71-3. The effect of benzalkonium chloride was also potentiated by preexposure to the furanone. Because the mechanism of action of the two disinfectants tested is different, it is likely that the observed enhanced effect by the furanone is linked to interference with a general protection mechanism of the bacterial cells, such as biofilm and not to a specific intrinsic resistance mechanism.

The effect of the furanone was probably associated both with the build-up of biofilm matrix and biofilm architecture. By treating the biofilm with furanone alone, no statistically significant reduction in the number of CFU was observed for either of the two strains tested. For this reason, our results indicate that this furanone affects the matrix in the biofilm, thus making the biofilm enclosed cells more susceptible to subsequent antimicrobial treatment. Results from the crystal violet colorimetric assay showed that one mode of action of the furanone was reduction in biofilm build-up. This effect was seen in seven of the strains tested, including 2168-10, but not for the four remaining strains including 71-3. However, the potentiated effect of the two disinfectants with preexposure to furanone was observed on representatives from both groups, 2168-10 and 71-3, although significantly less evident for 71-3. This indicates an additional mode of action of the furanone, e.g. a change in matrix architecture or composition. The biofilm matrix affords a protection against, e.g. disinfectants to bacterial cells within the matrix (Lapidot *et al.* 2006). It is reasonable to believe that both a change in architecture, composition and reduction in biofilm build-up will influence this protection. Similarly, changes in biofilm architecture was also observed when treating *Pseudomonas aeruginosa* and *Escherichia coli* biofilms with a halogenated furanone compound (Ren *et al.* 2001; Hentzer *et al.* 2002).

Quorum sensing has been suggested to be important for biofilm formation and maturation by several different bacterial species, such as *Ps. aeruginosa*, *E. coli* and streptococci (Davies *et al.* 1998; Costerton *et al.* 2007; Lönn-Stensrud *et al.* 2007; Li *et al.* 2007). Brominated furanones have been reported to inhibit bacterial colonization and biofilm development through interference with quorum sensing pathways (de Nys *et al.* 2006). The furanone chosen in our study has previously been shown to have an inhibitory effect on AI-2 and biofilm formation in oral streptococci and *Staph. epidermidis* (Lönn-Stensrud *et al.* 2007, 2009). In our study, the AI-2-deficient *luxS*-inactivated mutant LT2-3819 was included as a control. No statistically significant difference was found in biofilm production by the LT2 *luxS* mutant and the LT2 wild type strain, indicating that *luxS* might be irrelevant in biofilm production. Furthermore, our results gave no indications that the *luxS* gene was involved in the furanone response observed, as the amount of biofilm was reduced in the presence of the furanone in both the LT2 *luxS* mutant and the LT2 wild type strains. When studying all 11 strains, neither biofilm mass observed without furanone nor the effect of furanone on biofilm build-up were correlated to the amount of AI-2 measured in the supernatants. This indicates that the furanone tested did not affect the biofilm through interfering with the AI-2 system. Similar results have been obtained when studying the effect of another furanone on *Salmonella* biofilm (Janssens *et al.* 2008). AI-2 is produced and responded to by *Salmonella* (Surette and Bassler 1998), but the role of this molecule in biofilm formation by *Salmonella* is debatable. The only genes shown to be regulated by AI-2 are genes involved in the ABC transporter system (*Isr* operon) responsible for the uptake of AI-2 (Taga *et al.* 2001). Wintzer *et al.* raised the question whether AI-2 is used as a signalling molecule in all bacteria or if it in some bacteria is released as a waste product or used as a metabolite (Winzer *et al.* 2002a,b;). On the other hand, a few publications have suggested that the *Salmonella luxS* gene plays a role in biofilm production (Prouty *et al.* 2002; De Keersmaecker *et al.* 2005). Through which mechanisms the furanone affects *Salmonella* biofilm is therefore still unknown. Consequently, more research is needed to establish the complete mode of action of the furanone along with studies on optimizing the use of the furanone for a more practical approach, e.g. simultaneously combining furanone and disinfectants. The use of synthetic furanones in the fight against *Salmonella* in natural environments might also have a different concern; natural biofilms are usually multi-species biofilms and the furanone may affect other bacteria differently than *Salmonella*, e.g. increased biofilm formation by other micro-organisms like in the case of *Listeria monocytogenes* (Sela *et al.* 2006).

In conclusion, the present study showed that preexposure of *Salmonella* biofilms with a brominated furanone before disinfection with the conventional disinfectants hypochlorite and benzalkonium chloride potentiated the bactericidal effect of the disinfectants. This is valuable new knowledge in the fight against *Salmonella* in the environment although more information regarding the mode of action and practical approaches of the furanone in environmental settings is needed.
